# Over 100 Years of Rift Valley Fever: A Patchwork of Data on Pathogen Spread and Spillover

**DOI:** 10.3390/pathogens10060708

**Published:** 2021-06-05

**Authors:** Gebbiena M. Bron, Kathryn Strimbu, Hélène Cecilia, Anita Lerch, Sean M. Moore, Quan Tran, T. Alex Perkins, Quirine A. ten Bosch

**Affiliations:** 1 Quantitative Veterinary Epidemiology, Wageningen University and Research, 6708 PB Wageningen, The Netherlands; 2 Department of Biological Sciences and Eck Institute for Global Health, University of Notre Dame, Notre Dame, IN 46556, USA; kstrimbu@nd.edu (K.S.); alerch2@nd.edu (A.L.); smoore15@nd.edu (S.M.M.); qtran4@nd.edu (Q.T.); taperkins@nd.edu (T.A.P.); 3 BIOPAR, INRAE, Oniris, 44300 Nantes, France; helene.cecilia@oniris-nantes.fr

**Keywords:** bunyavirales, mosquito-borne disease, notifiable disease, Rift Valley fever phlebovirus, ProMED

## Abstract

During the past 100 years, Rift Valley fever virus (RVFV), a mosquito-borne virus, has caused potentially lethal disease in livestock, and has been associated with significant economic losses and trade bans. Spillover to humans occurs and can be fatal. Here, we combined data on RVF disease in humans (22 countries) and animals (37 countries) from 1931 to 2020 with seroprevalence studies from 1950 to 2020 (n = 228) from publicly available databases and publications to draw a more complete picture of the past and current RVFV epidemiology. RVFV has spread from its original locus in Kenya throughout Africa and into the Arabian Peninsula. Throughout the study period seroprevalence increased in both humans and animals, suggesting potentially increased RVFV exposure. In 24 countries, animals or humans tested positive for RVFV antibodies even though outbreaks had never been reported there, suggesting RVFV transmission may well go unnoticed. Among ruminants, sheep were the most likely to be exposed during RVF outbreaks, but not during periods of cryptic spread. We discuss critical data gaps and highlight the need for detailed study descriptions, and long-term studies using a one health approach to further convert the patchwork of data to the tale of RFV epidemiology.

## 1. Introduction

Over 100 years ago, in June 1912, an outbreak of “an obscure disease [that] caused heavy mortality in lambs” was described which, temporarily, had a discouraging effect on the sheep industry in Kenya [1]. Eighteen years later, in 1930, the likely causative agent, Rift Valley fever virus (RVFV), was isolated by Daubney and colleagues [2]. Nearly 100 years later, this primarily mosquito-transmitted virus, now known as Rift Valley fever phlebovirus [3], still causes morbidity and mortality in animals with spillover to people throughout the African continent, Indian Ocean, and Middle East. Outbreaks can lead to severe recurring economic losses, disrupting the livelihoods of often poor communities. Due to its economic impact, pathogenicity, and unpredictable (re)emergence, RVFV is recognized as a danger for both human and animal populations [4]. A better understanding of the eco-epidemiology of RVF could help inform intervention and surveillance strategies to reduce the burden of disease and minimize pathogen range expansion.

In the face of climate change, range expansions and redistributions of mosquito-borne viruses are expected [5]. RVFV, mostly transmitted by *Culex* and *Aedes* spp. mosquitoes, first expanded its range outside Africa in 2000, when it caused major outbreaks on the Arabian Peninsula [6,7,8,9]. The suitable geographical ranges for competent mosquito and ruminant populations that are conducive to pathogen introduction and spread are changing, alarming Europe and the Americas [10,11]. Historically, the emergence of RVFV in new areas has been unpredictable, but it has frequently been linked to animal trade [12,13,14]. As “a transmissible disease that has the potential for very serious and rapid spread, without regards for national borders, and with serious socio-economic and public health consequence as well as major importance in the international trade of animals and animal products,” the World Organization for Animal Health (OIE) recognizes RVF as a notifiable animal disease of concern. Similarly, the World Health Organization’s research and development blueprint named RVFV a priority pathogen due to its “epidemic potential” [4].

The impact and disease burden of RVF on local economies and livelihoods exemplifies the potential devastating consequences that RVFV could have on the global food supply and population health. Outbreak sizes in humans have been estimated to range from a few cases to thousands of cases, with case fatality risk ranging from 1 to 30% [15]. Human cases—ranging from mild to flu-like symptoms to hemorrhagic fever and death—are most common in individuals with close contact with animals and those consuming raw meat [16], often in rural communities. In Africa, more than 740 million individuals are currently living in rural, mostly agricultural communities, and this is expected to increase to 1039 million by 2050 [17], placing them and their animals potentially at risk for RVF. Outbreaks in animals are recognized by abortion storms and high mortality in young animals [2]. Although sheep and goats are most susceptible, RVFV also affects cattle, camels, and, sporadically, wildlife [18,19]. No RVFV treatments are registered for humans or animals, and medical treatments, when available, are limited to supportive care.

The strategies for RVF outbreak prevention and control are limited [20]. At a local level, communities and livestock owners could implement strategies to reduce mosquito bites, e.g., mosquito-control, moving animals to high-elevations pastures during peak mosquito seasons. In addition, individual animals can be protected and herd immunity can be accomplished by vaccinating animals [21,22]. However, preventative vaccination can be challenging due to a low burden of disease in the absence of outbreaks, and logistical and economical barriers, in part due to limitations of the currently available vaccines. For example, the most commonly used vaccine in livestock is a live-attenuated vaccine (i.e., Smithburn vaccine) which is highly effective [23] but can cause abortion and birth defects when vaccinating pregnant animals [24]. When RVFV is suspected in animals, control measures include, for example, announcements for hygiene measures, awareness, and vaccination campaigns [25]. However, when cases are confirmed in humans, a widespread outbreak is generally suspected and more expensive and intense measures are used, ranging from vector control to ruminant trade bans [25,26]. These interventions can disrupt local, regional, and national economies. For example, the estimated economic impact of a RVF outbreak from 2006 to 2007 ranged from 0.01% of the gross domestic product (GDP) in Tanzania (6.7 million US$) to 5.5% of the GDP in Somalia (471 million US$), making outbreak prevention a potentially more cost-effective strategy [27,28]. To strengthen RVF outbreak prevention, predictive modeling could be used to facilitate early community communication, targeted mosquito control interventions, and even localized vaccination campaigns [29].

RVF outbreaks in animals and humans are associated with a variety of factors, but the eco-epidemiology is not fully understood. RVFV circulates through different transmission routes;infection may occur through an infectious mosquito bite, or by contact with infected tissues and fluids (e.g., aborted tissues, exposure during slaughter). Outbreaks in animals, and subsequently in people, are often associated with increased rainfall in historically endemic areas, most notably the foci along the Great Rift Valley [30,31]. The virus, capable of vertical transmission, likely survives in *Aedes* spp. eggs in the environment. When infected eggs hatch during rainfall and flooding events, the virus might be able to re-emerge. Next, flooded areas provide ample breeding habitat for mosquitoes, further amplifying transmission. These patterns have been seen during, for example, the 1973–1975 South African, and the 1997–1998 and 2006–2007 Eastern Africa outbreaks, which were associated with El Niño rain events [32,33]. In other areas, RVFV may be introduced via the live-animal trade. Movements of infected animals from endemic regions with year-long presence of mosquitoes, can (re)introduce the virus to more seasonal ecosystems, in a source-sink fashion. Trade-related introductions and outbreaks are frequently associated with large gatherings, whereby large numbers of potentially infected animals are brought in for slaughter [12,34,35]. Despite the recognition of the underlying factors, the complexity of the dynamics together with a scarcity in data hamper actionable risk assessments.

To improve our understanding of the historic and current epidemiology of RVF, and to aid in planning future surveillance and intervention strategies, we compiled historical RVF data (including grey and white literature) on outbreak reporting and seroprevalence studies of both humans and animals. We examined patterns in the data associated with the occurrence of outbreaks and cryptic spread, including the seroprevalence in ruminant species, and examined if and how indicators of RVFV occurrence in animal populations are correlated to spillover to humans. As the data used largely come from outbreak reporting, which is highly heterogeneous across regions and over time, one should be careful in drawing hard conclusions from these analyses. Rather, this work should be regarded as hypothesis generating and aims to identify research gaps and needs based on a comprehensive overview of different data sources on this emerging zoonotic disease.

## 2. Materials and Methods

To explore the temporal and spatial activity of Rift Valley fever virus (RVFV) in Africa and beyond, we combined human and animal (domesticated animals and wildlife) case records and serological studies. To create a dataset as complete as possible, we included information sources beyond peer-reviewed (experimental) studies (an integrative literature review) but refrained from expert consultation and interviews. We extracted data from case reports, outbreak reports, and serological studies available in openly accessible, online databases, and in peer-reviewed journals. We then explored relationships among these records. Mosquito records were excluded.

### 2.1. Data Acquisition Strategy

**Figure 1:**
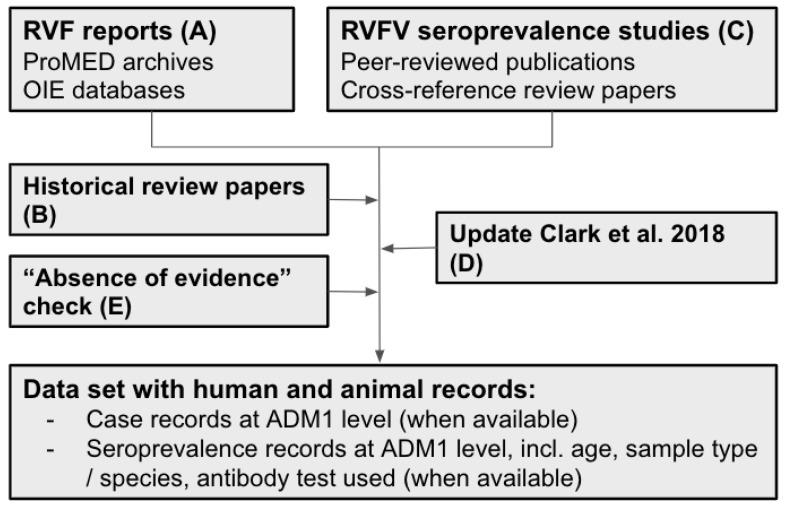
Flowchart of the source and data acquisition. A total of 275 sources ((**A**) two OIE databases and ProMED, and (**B**–**E**): 272 publications) were used to inform the RVF case and RVFV seroprevalence dataset for humans and animals. RVF reports were extracted from databases (**A**) and supplemented with historical review papers (**B**). Seroprevalence data was first extracted from publications identified through cross-referencing review papers (**C**) and the search strategy of Clark et al., 2018 was repeated to identify and add publications from 2017 to 2020 (**D**). In step (**E**), a search was conducted to explore if publications were available for African, Southern and Western Asian countries without RVF reports and RVFV seroprevalence studies to confirm absence of RVFV evidence. Data was included until 31 December 2020; the last update was conducted on 13 January 2021 by repeating step A and D. ADM1: Administrative Level 1, e.g., districts, provinces.

Data was extracted in five steps, A to E, by hand (Figure 1). First, we obtained RVF case data from databases on outbreaks in humans and animals (step A), complemented with data from systematic literature reviews (step B). Next, we extracted seroprevalence data from systematic literature reviews by cross-referencing the included studies (described in more detail later) (step C). We updated existing reviews to the current date (step D) and confirmed the absence of RVFV evidence for selected countries (step E, Figure 1). The database was last updated on 13 January 2021, and includes publications published up until 31 December 2020, including data pertaining to 2020 and earlier.

In step A, RVF case records were extracted from posts from the Program for Monitoring Emerging Diseases (ProMED) from the International Society for Infectious Diseases (ISID) and the public databases from the World Organization for Animal Health’s (OIE). ProMED was established in 1994 and archived posts were accessible from 1996 onward [36]. The OIE’s Handistatus 2.0 database [37] contained data from 1995 to 2004, after which it was replaced by the current World Animal Health Information System (WAHIS/WAHID) interface [38]. RVF case data were compared against the World Health Organization’s Rift Valley fever outbreak bulletins [39] and the Centers for Disease Control and Prevention (CDC) outbreak summaries [40] from 2000 onward to ensure no human RVF cases were omitted. Next, in step B, additional case records were extracted from historical review papers and reports [33,41,42,43,44].

To identify and extract data from original seroprevalence studies, we built on existing systematic literature reviews [45,46], and cross-referenced references within these publications (step C). Hereby, we included all geographical regions, e.g., studies from outside Africa and the Arabian Peninsula were included. Next, we used the search strategy from the most recent RVFV seroprevalence review, Clark et al., (2018) [45], to include publications published after 2016. Using the search term ((“Rift Valley Fever” OR “rvf”) AND (“prevalence OR “incidence” OR “sero)) in PubMED and Web of Knowledge, 102 and 162 publications were identified, respectively. There were 88 duplicates between the two searches. Of the 176 unique publications, 34 were already in our dataset (i.e., obtained through cross referencing); 88 were excluded based on their title (e.g., not relating to RVF); for 54 publications, the abstract was assessed after which 21 were excluded as they did not contain information pertaining to RVF or did not include original or new data, and data from 33 publications were added to the dataset. In addition, we conducted an absence of evidence search (step E) to explore if publications were available for countries from African, Southern and Western Asian United Nations (UN) regions [47] with neither serological nor outbreak data (Google Scholar search terms: (“*country name*”, “Rift Valley fever virus”, serology)). For 30 countries, no RVFV data was found, including six countries on mainland Africa: Algeria, Burundi, Eritrea, Guinea Bissau, Lesotho and Liberia.

### 2.2. Dataset

Information parsed from the different sources was summarized in four data types: human RVF cases, animal RVF cases, human serology, and animal serology.

#### 2.2.1. RVF Case Definition

Case definitions for RVF vary between agencies, reporting countries, and reports. As such, we did not use a standardized case definition and included any reported and suspected cases. Broadly, a case is defined as an individual infected with RVFV, with or without clinical symptoms. Cases of RVF in animals are often recognized by clinical symptoms in the herd but may go unnoticed. Determining the number of infected animals is therefore challenging. The OIE includes records on suspected transmission without case confirmation in their HandiStatus 2.0 and WAHIS database. We included those entries in our animal RVF dataset as well. In contrast, a human case of RVF is often defined as an individual with moderate to severe clinical symptoms who sought medical care. For both animals and humans, case numbers were included in the datasets, when available, but again, it is important to note that these cases represent varying levels of certainty. Cases were not always associated with a confirmatory diagnostic test. Speculations by authors on the total outbreak size were not included as cases, but these estimates were noted in a ‘notes’ column in the database (Supplementary Table S1).

A year with RVF cases was defined as any calendar year for which positive case numbers in humans or animals were reported or suspected for a country. We used this term synonymously with RVF outbreak, as an outbreak year was defined as any calendar year for which case data were reported. Laboratory-based cases were not considered. Inter-epizootic cases were included, whereas travel-related cases were excluded. Sometimes, an outbreak year only had one reported case. RVF outbreaks that spanned two calendar years (e.g., November to February) were marked in both years. When data sources reported a year from July to June (sometimes referred to as collating by season) and no additional information was available about the time of the observation (i.e., month or week), we also marked the observations in both calendar years.

#### 2.2.2. Geographical Information

Countries were grouped according to UN geographical regions [47]. The Canary Islands (ES-IC) were grouped with Northern Africa. Countries were abbreviated using three-letter codes and, when possible, information was parsed to administrative level 1 (ADM1) using the regions, districts, and provinces included in the Database of Global Administrative Areas (GADM) [48]. ADM2 and other smaller locations referenced (e.g., farm names) were collated at the ADM1 level. Since our database spans 91 years, including the period of decolonisation in Africa, when many nations (re)gained their independence, country names changed, borders were adjusted, and new nations were formed. To address this, we matched countries with the current UN and GADM database (e.g., Rhodesia, now Zimbabwe). In addition, historic RVF cases and serological studies may have taken place in an area of a country which is now recognized as an independent country by the UN. We used the current country names based on ADM1 level information or general regional directions of the data (e.g., South Sudan). Hence, older studies can be linked to countries that were not formally established at the time the study was conducted.

#### 2.2.3. Serological Records

Reports on the presence of RVFV antibodies in humans and animals were summarized by country (ADM0), ADM1 (when available), year(s) of sample collection (if this information was not available, authors were contacted), the number of individuals tested, number positive, and study sample characteristics. Data were further characterized including the age of human participants, animal species, or people-sampling strategies (e.g., random sampling of a general population; sampling of febrile, hospitalized or suspected patients; testing of high-risk individuals; unclear strategies or mixed samples), the type of antibody test used (e.g., enzyme-linked immunosorbent assay, immunofluorescent antibody test, virus neutralization test, complement fixing, plaque reduction neutralization tests), and antibody type targeted. If both IgM and IgG results were available, we included the IgG results and marked IgM availability in the notes. It is important to note that the presence of IgG antibodies does not mean the virus is circulating in the area where the sample was taken, and that movements and vaccination status of individuals should be accounted for. Cross-reactivity with other circulating viruses may also interfere with the interpretation of seroprevalence estimates, depending on the test used (e.g., African phleboviruses cross-reacting in haemagglutination-inhibition assays). In areas without known cases, the presence of RVFV should be confirmed, and paired sera should, ideally, be taken to confirm seroconversion and thereby recent exposure.

### 2.3. Statistical Analyses

Summary statistics regarding the temporal and spatial extent of each of the datasets are presented first. To further explore the aggregated data at the country and year level, we assessed how RVFV seroprevalence, and case data of animals and humans were associated to each other in regression analyses.

#### 2.3.1. Data Preparation

For our statistical analyses, we aggregated data at the country level, because for about a quarter of the entries, ADM1 was unknown (394 of 1507 records). Seroprevalence data were cleaned prior to analysis, excluding IgM-only records, records with missing data (e.g., study year or exact number of positives), and data points that could not be categorized as either outbreak- or non-outbreak-associated (e.g., data points reporting multiple years only partly overlapping an outbreak). A serological record was considered associated with RVF outbreaks if the samples were taken during the year in which RVF cases were recorded in humans or animals in the same country, or in the year after. Here, we made the assumption that post-outbreak serology is typically performed in the outbreak region. We test this assumption on the subset of data for which sufficient information is available. For those instances that this cannot be verified, we discuss the impact of the assumption. We limited outbreak-associated serology to those surveys performed up to one year post-outbreak.

#### 2.3.2. Annual RVF Case and Serological Study Availability

We estimated the increase in annual data availability for the four data types to quantify the progress made in data availability over time. For each data type, the response variable denoting if RVFV cases or a serological study was available in a given year (1) or not (0) was regressed against year: 1930 to 2020 for RVF cases and 1930 to 2017 for serological studies. We report the estimated slope of the logistic regression models.

#### 2.3.3. Seroprevalence over Time, and across Regions and Outbreaks

We compared seroprevalence between samples that were or were not associated with recent RVF cases (outbreak-associated: yes or no) using logistic regression models weighted by sample size. In these models, we accounted for differences between geographic regions (reference level: Eastern Africa) and over time. Time was rescaled to 10-year time steps. The start year of sample collection was used when a study reported multiple sampling years combined. The logistic regression analysis of human seroprevalence data was conducted for a subset of data including randomly sampled individuals only. The regression analysis of animal seroprevalence data was conducted for the full dataset and a subset of data including ruminant samples only (including camels, but excluding mixed samples with horses, mixed wildlife samples, and donkey, pig and rodent samples). Model estimates were exponentiated to calculate the adjusted odds ratios (aOR) of finding a positive sample in a group compared to the reference group.

#### 2.3.4. Ruminant Species’ Seroprevalence and the Association with Reported Outbreaks

To further dissect possible species contribution to RVFV, the logistic regression was repeated on a subset of data including only records for single ruminant species (sheep-reference level, buffalo, camels, cattle, goats). The species model accounted for differences in time (i.e., the year of sample collection) and geographical region (reference level: Eastern Africa), as above (Section 2.3.3), and assessed the association of species, recent RVF cases (yes or no), and their interaction with seroprevalence. The model was weighted by sample size. Variance inflation factors were assessed (<3 was accepted). Model estimates were exponentiated to calculate aOR. The interaction between species and outbreak was displayed using aORs with sheep in the absence of an outbreak as the reference level. The likelihood of detecting a positive buffalo, camels, cattle, and goats in the absence of an outbreak was calculated by exponentiating the model estimates. The effect of an outbreak on seroprevalence of sheep was calculated by exponentiating the outbreak estimate. The aORs for the other species during an outbreak were calculated by exponentiating the sum of three model estimates: the estimates for the species (1), outbreak (2) and the interaction of the species and outbreak (3).

#### 2.3.5. Exploring Cryptic Spread: RVFV Activity Prior to RVF Case Reporting

To assess if cryptic RVFV transmission may be present (i.e., animals were exposed to RVFV, but no cases were reported), we extracted all seroprevalence data when RVF had never been reported in the country. We compared, by ANOVA, if the mean seroprevalence (by country and year) was different based on the current, 2020, RVF status of the country (i.e., RVF cases have been reported since the seroprevalence study was conducted, or no cases have been reported to date).

#### 2.3.6. Exploring Pathogen Spillover: Concurrent Animal and Human RVFV Activity

To assess if animal case investigations were more likely when human RVF outbreaks occurred, we compared, by Fisher’s exact test, if the proportion of animal case records with case counts was different when human outbreaks had or had not occurred in the same year and country. To determine if countries with higher seroprevalence in animals were more likely to have higher human seroprevalence, we summarized seroprevalence by country and year for randomly collected human samples and ruminant animal samples and assessed the Spearman’s rank-based statistic. As above, we acknowledge that the spatial scale of RVF outbreaks is typically smaller than the country level. We further examine the limited number of studies for which ADM1-level information is available for both human and animal studies to answer two questions: (i) how likely are studies in humans and animals from the same year to have been performed in the same ADM1, and (ii) for those records, can we distinguish patterns between human and animal seroprevalence and are those consistent with conclusions from the larger dataset?

#### 2.3.7. Software

All statistical analyses were conducted in R Statistical Computing Software [49]. Package *lme4* [50] was used for regression models. Figures were created using packages *rnaturalearth* [51] and *ggplot2* [52], and organized using *cowplot* [53].

## 3. Results

Information from OIE databases and ProMED archives was supplemented with data from 273 peer reviewed publications (Table 1). The dataset consists of 1507 records of four data types: 125 on human RVF cases extracted from 59 sources, 415 on RVF cases in animals from 38 sources, 294 on human serology from 106 sources, and 673 records on animal serology extracted from 144 sources (Supplementary Table S1).

### 3.1. Data Availability

#### 3.1.1. Data Availability over Time

**Figure 2:**
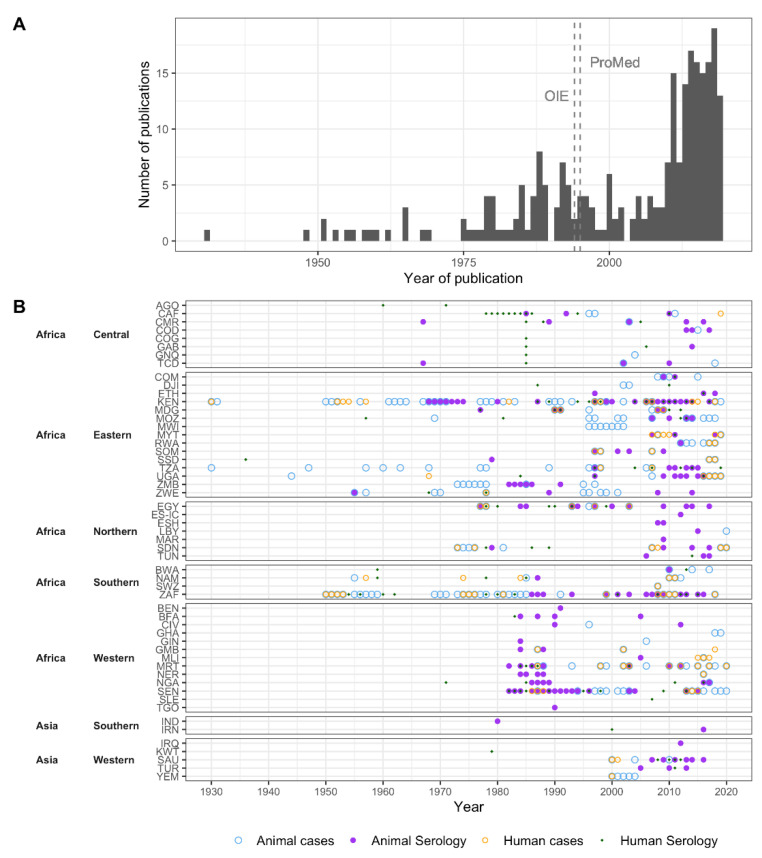
Regional variation in RVF reports and seroprevalence studies over time. (**A**) Number of included publications by year. Vertical lines represent the first year during which OIE and ProMED reports were included. (**B**) For each country, the years during which Rift Valley fever cases were reported, and the years during which Rift Valley fever virus seroprevalence data were collected are marked for animals and humans. When results from multiple years were reported as one, e.g., 1991–2000, we marked the first year in the figure. Serological data also include studies where no RVFV antibodies were detected; note that this is not a RVFV detection chart.

A total of 91 years were included in the dataset (1930 to 2020), with the first publication in 1931 [2] (Table 1, Figure 2). Publications on human and animal RVF cases were first available in 1931. The first publication on human seroprevalence estimates was published in 1956 [55], and on animal seroprevalence estimates in 1958 [56]. The average lag between study-end year and publication year of the manuscript for seroprevalence studies was three years for both human and animal studies. RVF cases that occurred prior to 1990 often had a lag in their publication, as case records were published in peer-reviewed sources (e.g., historical reviews, compared to current near real-time reporting). During the 1990s, this transitioned to predominantly same-year reporting. Over 40% of human and animal RVF reports we extracted came from archived ProMED posts (37 of 89 human, and 125 of 279 animal RVF records published after 1996, respectively), which were mostly reported the same year as the outbreaks occurred.

The number of RVFV publications increased over time (Figure 2A). For all four data types, the probability of there being at least one record in a given year increased significantly (*p* < 0.001), although the rate of increase differed between data types. This increase in publications was steepest for animal serology (beta = 0.124, 95% Confidence Interval [CI]: 0.082, 0.184) in comparison to human serology (beta = 0.089, 95%CI: 0.058, 0.129), human RVF cases (beta = 0.054, 95%CI: 0.033, 0.079), and RVF cases in animals (beta = 0.064, 95%CI: 0.038, 0.098) (Supplementary Figure S1).

#### 3.1.2. Spatial Distribution of Data

**Figure 3:**
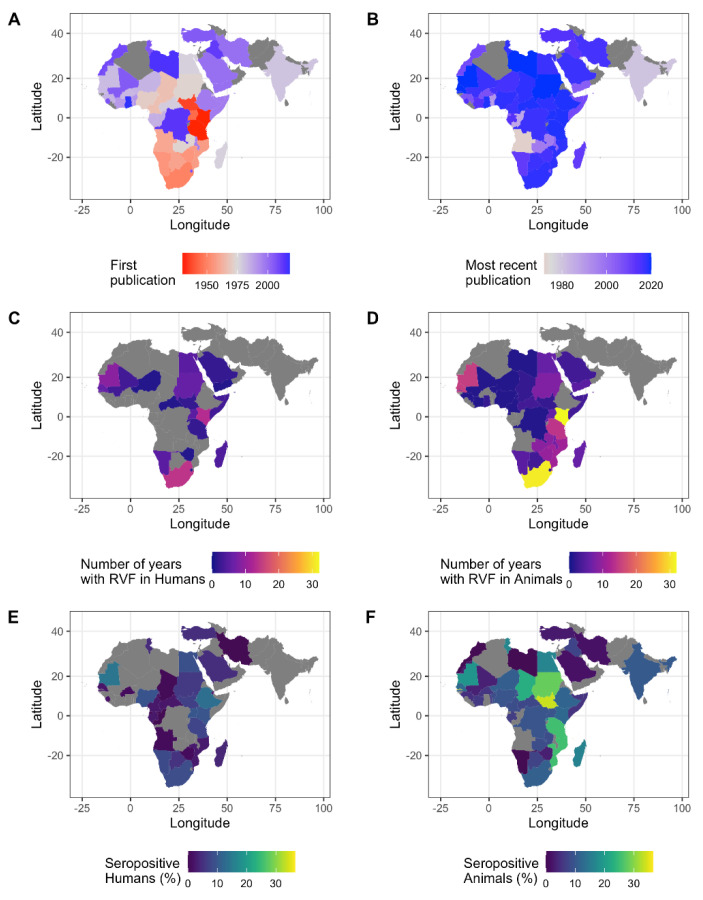
RVFV reporting and activity. (**A**,**B**) The year of the first (**A**) and most recent (**B**) RVFV publication by country. The studies on the Canary Islands, Comoros Islands, and Mayotte are not shown. No publications were found for the countries in grey. (**C**,**D**) Number of years with human (**C**) or animal (**D**) RVF case records per country. (**E**,**F**) Percent of individuals positive for RVFV antibodies of all individuals sampled per country for human samples (**E**) and animal samples (**F**). All samples, except IgM-only records, were included. Gray indicates that no RVF case reports or seroprevalence studies were found for this country. It should be noted that the presence of individuals with a positive RVFV antibody test does not ascertain local circulation of the virus and should be interpreted with caution, particularly for countries with no confirmed cases.

All five African regions, and about 80% of African countries, were represented in the dataset and thus had either RVF cases reported or seroprevalence studies conducted (Figure 3). In addition, Southern and Western Asia were included with two (Iran and India) and five countries (Saudi Arabia, Yemen, Iraq, Kuwait, and Turkey), respectively (Figure 3). Of the 52 countries in the dataset, human and animal RVF case records were found for 22 and 37 countries, respectively (Figure 3C,D). Human and animal seroprevalence studies were conducted in 33 and 45 countries, respectively (Figure 3E,F). Most human and animal serological publications (*n* = 106 and *n* = 142) originated from Kenya (22 and 15) or South Africa (10 and 13), followed by Egypt (7 and 12). For 15 countries, both human RVF cases and human seroprevalence data were available. For twice the number of countries (31 countries), both animal RVF cases and animal seroprevalence have been reported.

### 3.2. RVF Outbreaks and Number of Cases Affected

A total of 605,005 animal and 10,944 human RVF cases were reported during 68 and 45 years, respectively (Supplementary Table S1). Animal and human cases were most often reported in Kenya (32 and 13 years, respectively) and South Africa (31 and 14 years, respectively) (Figure 3C,D). Most animal cases were also reported from Kenya (507,996, 84% of total cases), followed by Tanzania (38,167) and South Africa (19,543). Most human cases were reported from Sudan (2534), Egypt (1267) and Kenya (1187).

A total of 224 animal and 95 human RVF case records were available after aggregating data by country and year. For 152 of the 319 aggregated records, case counts were available (47.6%). Most of the records with case counts (126 of 152, 82.9%) occurred in 2000 or later. The number of RVF cases in animals ranged from a single animal succumbing to RVFV (e.g., an antelope in Senegal in 2020) up to estimates of 250,000 animals affected during the 1950 to 1951 outbreaks in Kenya. Human case counts also ranged from one individual to about 1500, but it was noted that an estimated 20,000 to 100,000 people may have been infected in South Africa in the 1950s.

### 3.3. Variation in Seroprevalence

**Figure 4:**
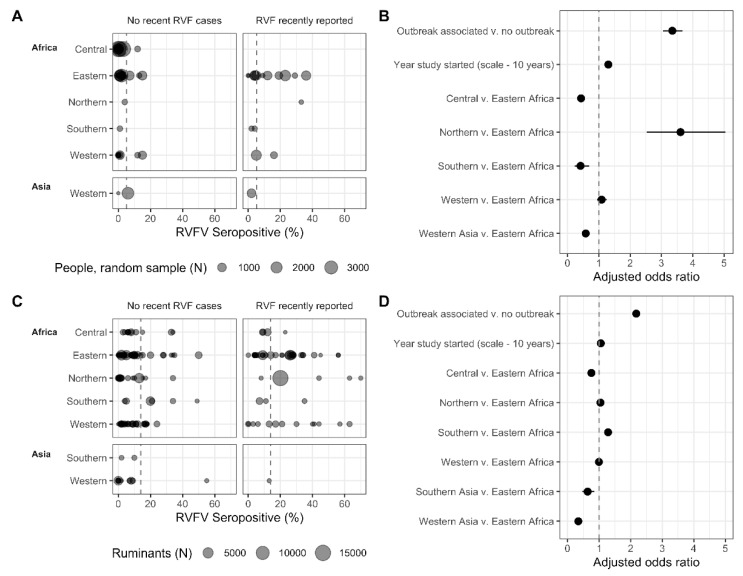
Regional human, individuals selected by random sampling, (**A**,**B**) and ruminant (**C**,**D**) RVFV seroprevalence in the absence of recent human and/or animal RVF cases or within the same year, or year post, reported RVF cases. (**A,C**) Sample size and seroprevalence per country and year. The grey, dashed vertical lines represent the mean seropositivity (**A**,**C**) and equal odds (**B**,**D**). Horizontal lines represent confidence intervals around the point estimate (**B**,**D**). Southern Asia is represented by studies from Iran and India; Western Asia is represented by Iraq, Saudi Arabia, Turkey and Yemen.

Over 250,000 individuals (human and animals) were represented in the seroprevalence data and 210,074 of which met the inclusion criteria: 131,378 animals and 80,406 humans. The percentage of individuals that tested positive for RVFV antibodies by country and year ranged from 0% (93 to 4590 individuals tested) to 40% (30 individuals tested) in humans, and 0% (1 to 1344 animals tested) to 70% (150 individuals sampled) in animals (Figure 4A,C).

#### 3.3.1. The Association of Seroprevalence with Time, Region, and Outbreaks

A subset with data on 48,702 randomly selected human individuals remained, after excluding those studies that targeted individuals with high-risk lifestyles and professions, febrile patients, and studies that had mixed or unclear sample selection. Similarly, 122,419 ruminant samples remained (including camels), after excluding studies with non-ruminants (e.g., rodents, horses, pigs, donkeys), and mixed samples of ruminants and non-ruminants (e.g., “pig and sheep”).

Among all the included samples, 5.2% of human samples and 13.9% of ruminant samples were positive for RVFV antibodies (Figure 4A,C). Of samples associated with an outbreak (i.e., human and/or animal cases in the same or previous year), 12.6% of human samples and 19.8% of ruminant samples were positive for RVFV antibodies. This was substantially lower for samples not associated with outbreaks, with 2.7% of human samples and 9.8% of ruminant samples positive for RVFV antibodies (Figure 4A,C). Indeed, when assessing the subset of randomly selected individuals, the adjusted odds (aOR) of finding a positive serum sample was 3.35 times higher (95%CI: 3.06, 3.67, *p* < 0.001) when sampling was conducted in the same year or the year after RVF cases were reported in the country compared to sampling conducted in the absence of recently reported RVF cases (Figure 4B). A similar, yet smaller effect was observed for ruminants, with aOR = 2.18 (95%CI: 2.10, 2.25, *p* < 0.001), indicating that more individuals are exposed to the virus during outbreak years than during cryptic cycles (Figure 4D). As expected, the proportion of individuals who tested positive for RVFV antibodies also varied over time and by region (Figure 4). Every 10 years, the probability for a sample to be positive increased 1.30 times (95%CI: 1.24, 1.37, *p* < 0.001) for the subset of randomly selected humans and, similarly, 1.05 times per 10-year timestep for ruminants (95%CI: 1.04, 1.07, *p* < 0.001) (Figure 4B,D). In addition, relative to Eastern Africa, the proportion of individuals who tested positive was higher in people in northern Africa, largely driven by a study conducted in an area affected by RVFV 13 years prior, (aOR: 3.61, 95%CI: 2.53, 5.04) and lower in individuals from central and southern Africa and western Asia (i.e., Kuwait, Saudi Arabia, Turkey) (aOR: 0.43, 0.41 and 0.58, 95%CI: 0.36, 0.51; 0.23, 0.69 and 0.49, 0.68) (Figure 4B, Supplementary Table S2). The probability for a ruminant being seropositive in central Africa, and southern (i.e., Iran and India) and western Asia (i.e., Iraq, Saudi Arabia, Turkey) was lower than in eastern Africa (Figure 4D, Supplementary Table S3). The seropositivity in the ruminant populations in north, west, and south African regions was similar to slightly higher than, eastern Africa. Observed patterns of time and outbreaks on the proportion of individuals who tested positive in ruminants were robust to using the full dataset (i.e., the dataset with non-ruminants included. Supplementary Table S4). For most records, it was not possible to verify if the seroprevalence studies were performed in the same ADM1-level as where the outbreaks occurred. About half of human outbreak records (65 of 125), and about a third of animal outbreak records (135 of 415 records) did not have ADM1-levels reported. When looking at the seroprevalence subsets for which ADM1-level were available (77.7% of randomly selected humans records (80 of 103 records containing 32,434 individuals), and 80% of ruminant records (410 of 515 records containing 79,112 individuals), and taking a conservative approach to considering a sample outbreak associated (i.e., if an outbreak occurred in the country, and the ADM1 was not known, the sample was not outbreak-associated), the general conclusions are robust, though effect sizes vary, and some regional associations changed (Supplementary Tables S5 and S6).

#### 3.3.2. Ruminant Species’ Seroprevalence and the Association with Reported Outbreaks

The percentage of animals testing positive varied between the different ruminant species sampled, with sheep having the lowest seroprevalence in the absence of recent RVF cases (Table 2). However, the interaction between species and outbreak-associated sampling was strongly significant (*p* < 0.001). While the odds of detecting a seropositive sheep in association with recent RVF cases tripled, the odds of finding positive buffalo, camels, cattle and goats only increased 1.26 to 1.48 times (Table 2, Supplementary Table S7). The higher odds of detecting a positive sheep in association with RVF cases indicates that this species could be more likely to be exposed during outbreaks, possibly amplifying the outbreak.

#### 3.3.3. Exploring Cryptic Spread: RVFV Activity Prior to RVF Case Reporting

**Figure 5:**
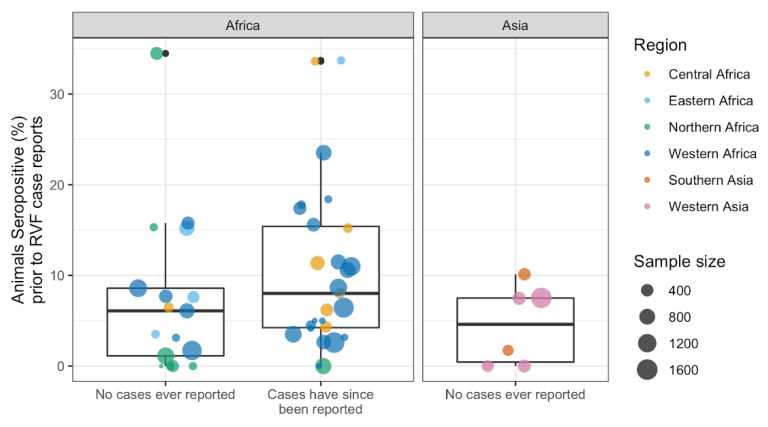
Rift Valley fever virus seroprevalence when RVF cases had never been reported in the country (human and/or animal). The outlier with no known RVF cases represents samples from Tunisiacollected between 2017 and 2018 (34.5% positive of 470 camels) [57], and two outliers currently known to report RVF cases represent samples collected in South Sudan from 1979 to 1983 (33.7% positive of 92 ruminants, cattle and goats) [58], and Cameroon in 1968 (33.6% of 122 sheep) [59].

Serological studies took place in 27 countries (including the Canary Islands) without known RVF cases, for a total of 50 aggregated records by country and year (Figure 5). In 24 countries, animals tested positive for RVFV antibodies; in 11 of these countries, RVF cases have never been reported to date, whereas in 13 countries, RVF cases were reported 1 to 38 years later. Notably, animals tested positive for RVFV antibodies in Iraq, Iran, and Turkey (Figure 3). However, confirmed animal or human cases have not been documented in these countries to date.

A total of 1859 of 24,642 animals tested positive for RVFV antibodies in countries without known RVF cases at the time of the survey (7.5%), 8.8% of animals from countries that later reported RVF cases (1217 of 13,788) and 7.2% of animals sampled in countries that, to date, have not reported RVF cases (827 of 11,454, Figure 5). No difference in seroprevalence, by country and year, was detected between the groups that currently report and do not report RVF cases (ANOVA, df 1,48, F = 2.5, *p* = *0*.12, Figure 5).

### 3.4. Exploring Pathogen Spillover: Concurrent Animal and Human RVFV Activity

#### 3.4.1. Association of Human RVF Outbreaks with Animal RVF Cases

There were 73 concurrent human and animal RVF outbreaks (i.e., human and animal cases reported in the same year and country); 77% of 95 human outbreaks and 33% of 224 animal outbreaks occurred concurrently. Concurrent outbreaks were reported in 21 counties, with most in South Africa (14), Mauritania (9) and Kenya (7).

For about half of the concurrent outbreaks, case counts were available for both humans and animals (38 of the 73 paired records). The human and animal case counts of concurrent outbreaks (38 paired records) were weakly positively correlated (Spearman S = 6588, rho = 0.279, *p* = 0.09, Supplementary Figure S2). The occurrence of human RVF cases increased the likelihood that case counts were available for animal RVF records: 59% (43 of 73) of concurrent outbreaks and 30% (45 of 151) of animal-only outbreaks had case counts (OR: 3.36, 95%CI: 1.81, 6.30, *p* < 0.001).

Of the 22 human RVF outbreaks without associated animal RVF cases, 16 occurred within two years of an RVF outbreak in animals, leaving six human RVF outbreaks without a link to reported animal RVF cases (6.3% of 96 human RVF outbreaks: 150 cases in Kenya from 2014 to 2015, 15 cases in the Central African Republic in 2019, one case in Gambia in 2018, and an unknown number of cases in Namibia in 1974 and Uganda in 1968).

#### 3.4.2. Correlation between Animal and Human Seroprevalence

A total of 17,269 people (selected using a random sampling strategy) and 11,777 ruminants were sampled in the same year and country. The 19 paired records of concurrent seroprevalence data include 10 countries (Kenya *n* = 9, Senegal *n* = 2, Central African Republic, Ethiopia, Madagascar, Mauritania, Mayotte, Tanzania, Uganda and South Africa) and 16 years (earliest 1982, most recent 2017).

**Figure 6:**
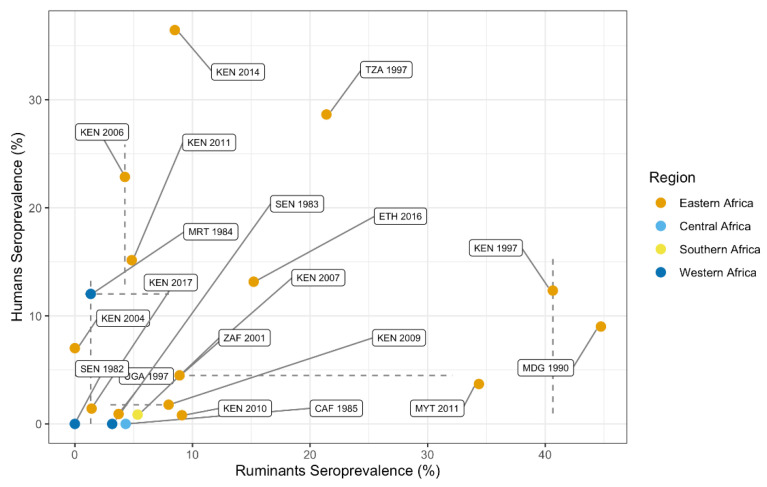
Animal and human RVF seroprevalence data from the same year and country. Point estimates represent mean seropositivity (percent of individuals testing positive of the total number of individuals tested) by country and year. The point estimates are color-coded by region. Lines represent the range in seroprevalence estimates reported when multiple publications were included in the prevalence estimate.

Countries with high seroprevalence in animals were not typically associated with high human seroprevalence during the same year (Spearman S = 720, rho = 0.368, *p* = 0.12, Figure 6). When considering the seroprevalence records that selected for high-risk individuals (e.g., herders, slaughterhouse workers), the association with ruminant seroprevalence was even weaker (12 paired country-year records, Spearman S = 120, rho = 0, *p* = 1). The absence of a significant correlation between human and ruminant seroprevalence may, among other factors, result from the fact that not all records originated from the same ADM1-level. ADM1-level information was available for human and ruminant seroprevalence records of 10 paired country-year records. Of those, three country-year records included the same ADM1 for animal and human serological data, a spatial scale which more precisely reflects RVFV outbreaks.

## 4. Discussion

Approximately 90 years of reporting, 70 years of surveillance efforts, and about 20 years of near real-time case report availability allowed us to map the pathogen’s geographic distribution, and the extent of co-occurrence of RVF in different host species. Explorations of this patchwork of different data sources allowed for the generation of new hypotheses on the epidemiology of RVF. For instance, analyses showed sheep are the most exposed species during outbreaks. This suggests they may play an important role in transmission of the pathogen, particularly during outbreaks. The dataset generated (Supplementary Table S1) provides the first open-access global human and animal RVF case and RVFV seroprevalence data compilation, and allows researchers to further investigate the epidemiology of the virus.

RVFV spread significantly over the past decades and appears to (re)emerge more frequently. For example, the pathogen spread to Saudi Arabia and Yemen from Africa in 2000. The pathogen was also successfully introduced to Madagascar, Mayotte and the Comoros Islands, with RVF outbreaks in recent years on both islands (2008 onward) [36,38]. Interestingly, evidence of RVFV circulation was described in 1977 in Madagascar, but the first cases were only reported in 1990. In addition, there is serological evidence of viral exposure in animals in Iraq [60], Iran [61] and Turkey [62] in the absence of RVF cases. However, as RVFV confirmation and animal travel histories are incomplete, local RVFV circulation cannot be established based on these records alone. Furthermore, the pathogen recently re-emerged in areas where no cases had been confirmed for over 40 to 50 years [63]. The increase in outbreak reports is in part due to improved reporting systems that are globally accessible, but not exclusively. Greater mobility and trade could facilitate the movement of susceptible and exposed animals. These movements in combination with increased densities of humans and animals, changing mosquito population dynamics, landscapes and weather patterns could all contribute to faster introductions and subsequent transmission. Indeed, animal and human exposure to RVFV appears to have increased over time. The approximate 30% increase in seropositivity in humans and 5% increase in animals every 10 years (e.g., 5% seroprevalence becomes 6.5% or 5.25%) suggests increased exposure to the virus. However, these changes in seropositivity could in part be due to improved, more sensitive diagnostic tools, and changed sampling strategies, whereby cross-sectional studies screening for hemorrhagic fever viruses were replaced by RVFV-specific studies in areas with known or suspected virus activity (e.g., outbreak investigations, intervention evaluations). In addition, an increase in seroprevalence is to be expected in longer-lived species, such as humans, even if the force of infection is relatively stable over time, due to the accumulation of exposed individuals who survived infection (i.e., seropositive individuals). Age-stratified sampling could help disentangle these historical transmission patterns [64], but few studies reported on the age of participants and fewer provided age-stratified data (for examples, see [65,66,67]). In addition, structured, long-term seroconversion studies that include both humans and main animal hosts could help provide local evidence of the force of infection and how it changes over time.

Our analyses corroborated a prominent role of sheep during outbreaks, finding them to be the most likely species to seroconvert during outbreaks, three times more likely than during cryptic transmission cycles. In comparison, goats were approximately 1.5 times more likely to seroconvert. This aligns with the belief that sheep are the most important host species for RVFV amplification, owing to their high susceptibility and viral loads [2,68]. These characteristics, in addition to sheep experiencing the most severe pathology due to RVFV, could make them the prime vaccine target to minimize economic losses for farmers and to prevent RVF outbreaks. Understanding the species’ local contribution to RVFV maintenance and amplification is important for vaccination and surveillance strategies to prevent RVF outbreaks.

Despite several large RVF outbreaks in animals, human case counts remained relatively low, suggesting either limited or geographical variation in spillover, or infections going unrecognized, undiagnosed, or unreported. In people, the probability of asymptomatic infection is estimated to range from 90 to 98% [69,70]. In addition, health care access and diagnostics may be limited, and RVF symptoms overlap with those of other prevalent diseases [71], possibly obscuring case identification and reporting. Similarly to humans, animal RVF cases may also be overlooked, thereby facilitating cryptic transmission of RVFV. The absence of animal cases when human RVF cases were observed suggests that livestock cases also go unreported, or human cases were strictly mosquito-mediated (mosquitoes becoming infected through vertical transmission or by feeding on infected wildlife) or due to slaughter or raw wildlife meat consumption. In a herd, RVF is easy to overlook when few animals are affected, or few animals show signs of disease [72]. As suggested by the greater likelihood of having case counts for animals when human cases occur simultaneously, outbreak investigations among animals are often initiated after human cases have been detected. In addition, the presence of RVFV antibodies without animal or human RVF cases in a country could be explained by cryptic transmission, whereby cases may be mild enough or in small enough numbers to go unnoticed. Some of the presence of antibodies in the absence of cases could be explained by RVFV exposure at different geographical locations, i.e., in traded animals, and seroconversion due to vaccination. Overall, the evidence of cryptic transmission urges reconsideration of reliance on passive surveillance systems for the detection of RVFV, and other emerging pathogens.

Increased data availability, through historical reviews, studies sharing their (raw) data, and near real-time reporting of RVF cases facilitated the creation of this comprehensive dataset, thereby connecting the patchwork of RVFV data and improving epidemiological inference made in these publications. Our study highlights the value of detailed reviews at a national level that summarize historic local and national database records—many previously unavailable—with as much detail as possible (for example, [41,43]). Furthermore, our dataset illustrates the importance of centralized databases, as many of RVF case records were sourced from ProMED-archives and the OIE. Notably, ProMED combines grey literature with official reporting of cases and outbreaks to international organizations, expanding its reach beyond traditional data sources. We thus purposely expanded our search strategy to go beyond peer-reviewed published (experimental) studies (an integrated approach, e.g., including theses, ProMED, OIE databases), used a broad case definition, and accepted all serological tests detecting IgG. By adopting this strategy, we were able to picture a more complete view of the history of RVF epidemiology. However, the robustness of data and conclusions from the analyses should also be considered in this context. The information gained from patterns in the data should be further explored and investigated in more detail when additional data become available.

Standardization in reporting would strengthen the dataset and the inferences that can be made from the data in future analyses. For example, serological studies were inconsistent in reporting the importation and vaccination history of the animals, possibly inflating seroprevalence. Similarly, the migration and travel history of people was often not included, placing the location of RVFV antibody detection away from the true location of infection. In addition, although districts, regions or provinces of sampling were often reported, the data was not shared at these geographical levels in about a quarter of publications. Therefore, we summarized data by country and year to create a more robust dataset. This aggregation sacrifices the fine-scale information and prevents describing and detecting within-country variation. Since RVF outbreaks are often localized, they can prompt outbreak investigations and seroprevalence studies beyond the affected area; our aggregation of the dataset combined studies from areas with and without RVFV transmission, thereby possibly underestimating seroprevalence associated with outbreaks. Furthermore, we identified samples as outbreak-associated when samples were collected during the outbreak, or the year after the outbreak (reported RVF cases in humans and/or animals). This grouping potentially placed samples collected at outbreak locations two years or more after an outbreak in the non-outbreak group (e.g., sampling in Egypt 13 years post-outbreak [73]). Combined, this meant that our adjusted odds ratios likely underestimated the actual increase in human and animal seroprevalence due to RVF outbreaks.

As the geographical range suitable for RVFV introduction and establishment continues to expand, global efforts should continue to improve surveillance strategies to detect pathogen emergence and prevent pathogen spread. Similarly, with the increasing frequency of outbreaks, both in eastern and western Africa, regional and national programs may adjust their disease surveillance and RVFV intervention program. To evaluate if RVFV activity and exposure is increasing, as suggested by the increased seroprevalence over time, targeted long-term studies in endemic and non-endemic areas could be started. These efforts would be more beneficial if a One Health approach were to be used [74], in which human, animal, and mosquito populations, as well as the environment, are monitored simultaneously. Looking ahead, standardized reporting of serological studies, and uniform case definitions would ensure that on-the-ground, local efforts can be utilized at a larger geographical scale, further informing mathematical models and facilitating a thorough understanding of RVFV epidemiology. Ultimately, these research and reporting efforts combine local interests and international research priorities to limit the burden of RVFV.

## Figures and Tables

**Table 1:** Peer-reviewed publications by region. The median year of publication and range are included. The OIE databases (*n* = 2) and ProMED archive (available since 1996) are not included in this table.

Continent	Region	Sources	Year of Publication of Used Sources		
			Earliest	Median	Most Recent
Africa	Central	22	1965	2008	2019
	Eastern	102	1931	2013	2020
	Northern	35	1978	1999	2020
	Southern	33	1951	2011	2020
	Western	47	1980	2009	2020
Asia	Southern	2	1995	2000	2005
	Western	21	1984	2013	2020
Multiple and other regions *		11	1969	2011	2020
Total		273	1931	2011	2020

* One publication from Poland, Europe, is available [54].

**Table 2:** Species-specific seroprevalence and adjusted odds ratios for the interaction between species and recent RVF reporting.

Species	No Recent RVF Cases				RVF Recently Reported			
	%	(*n*)	aOR	95% CI	%	(*n*)	aOR	95% CI
Sheep	8.1	(17,328)	1	(Reference)	21.6	(10,271)	2.99	(2.77, 3.23)
Buffalo	13.8	(1417)	1.47	(1.25, 1.73)	15.7	(1523)	1.85	(1.16, 2.93)
Camels	10.0	(4938)	1.80	(1.60, 2.03)	20.5	(1451)	2.62	(1.80, 3.82)
Cattle	13.0	(22,288)	1.47	(1.37, 1.58)	18.2	(19,519)	2.12	(1.67, 2.69)
Goats	11.9	(8746)	1.24	(1.14, 1.35)	17.1	(5968)	1.83	(1.38, 2.43)

Common livestock species were compared to sheep tested when RVF had and had not been recently reported in a country. The logistic regression model also accounted for the year the study was started and the region the work was conducted in (Supplementary Table S7). aOR: adjusted odds ratio; CI: confidence interval.

## Data Availability

Data is contained within the supplementary material and the Open Science Framework (OSF) repository (DOI 10.17605/OSF.IO/UXRA6), as part of the OSF project (DOI 10.17605/OSF.IO/54UKB). Code to reproduce figures and tables is available in the OSF project (https://osf.io/54ukb/).

## Supplementary Materials

The following are available online at https://www.mdpi.com/article/10.3390/pathogens10060708/s1, Figure S1: Data availability over time (logistic regression plotted), Figure S2: Human and Animal RVF cases, Table S1: Dataset, Table S2: Seroprevalence model output–People, Table S3: Seroprevalence model output–Ruminant, Table S4: Seroprevalence model output-All animals, Table S5: Seroprevalence model output–People matched by ADM1, Table S6: Seroprevalence model output–Ruminant matched by ADM1, Table S7: Seroprevalence model output-Selected ruminant species
