# Supplementary figures and images for: Over 100 Years of Rift Valley Fever: A Patchwork of Data on Pathogen Spread and Spillover
Source: Pathogens. 2021 Jun 5;10(6):708. doi: 10.3390/pathogens10060708 (PMC8227530; doi:10.3390/pathogens10060708)

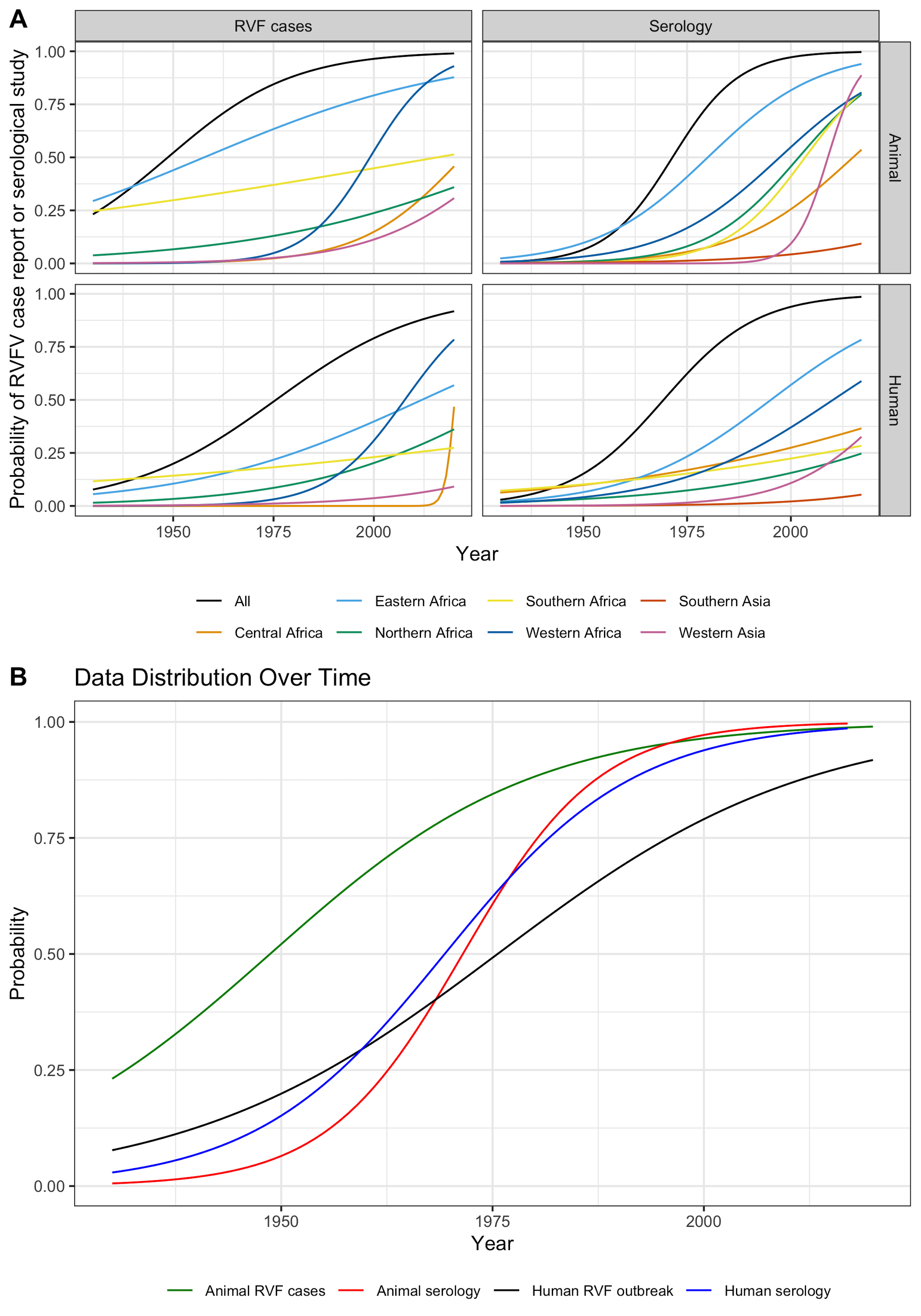

Supplement: Supplementary file 1 [file pathogens-10-00708-s001.zip › Supplemental Figure S1.tiff]

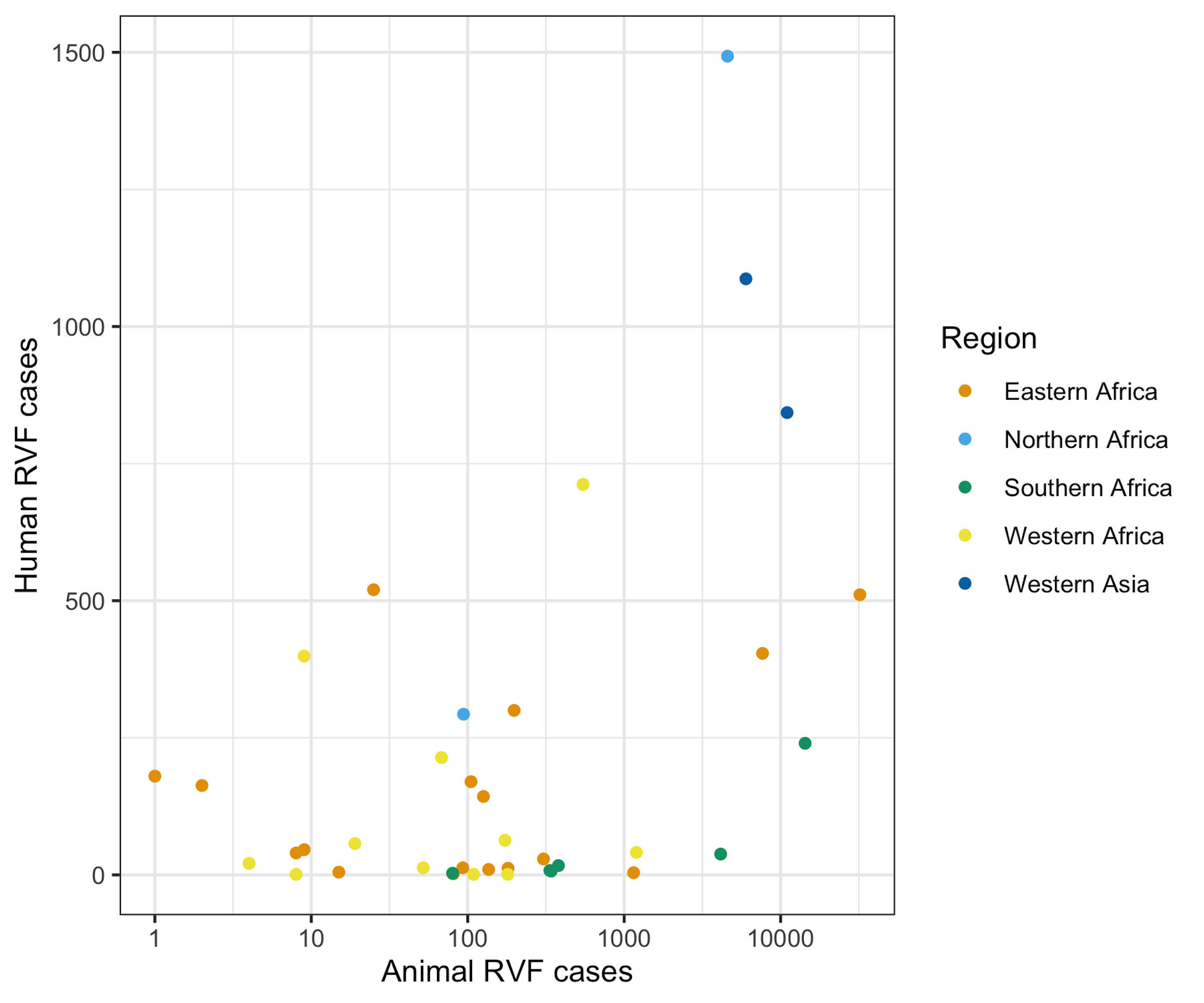

Supplement: Supplementary file 1 [file pathogens-10-00708-s001.zip › Supplemental Figure S2.tiff]
